# Reporter Gene-Facilitated Detection of Compounds in *Arabidopsis* Leaf Extracts that Activate the Karrikin Signaling Pathway

**DOI:** 10.3389/fpls.2016.01799

**Published:** 2016-12-02

**Authors:** Yueming K. Sun, Gavin R. Flematti, Steven M. Smith, Mark T. Waters

**Affiliations:** ^1^Australian Research Council Centre of Excellence in Plant Energy Biology, The University of Western Australia, PerthWA, Australia; ^2^School of Chemistry and Biochemistry, The University of Western Australia, PerthWA, Australia; ^3^School of Biological Sciences, University of Tasmania, HobartTAS, Australia; ^4^Institute of Genetics and Developmental Biology, Chinese Academy of SciencesBeijing, China

**Keywords:** karrikin, plant hormone, reporters, chemical biology, strigolactone, *Arabidopsis*, germination

## Abstract

Karrikins are potent germination stimulants generated by the combustion of plant matter. Treatment of *Arabidopsis* with karrikins triggers a signaling process that is dependent upon a putative receptor protein KARRIKIN INSENSITIVE 2 (KAI2). KAI2 is a homolog of DWARF 14 (D14), the receptor for endogenous strigolactone hormones. Genetic analyses suggest that KAI2 also perceives endogenous signal(s) that are not strigolactones. Activation of KAI2 by addition of karrikins to *Arabidopsis* plants induces expression of transcripts including *D14-LIKE 2* (*DLK2*). We constructed the synthetic reporter gene *DLK2*:*LUC* in *Arabidopsis*, which comprises the firefly luciferase gene (*LUC*) driven by the *DLK2* promoter. Here we describe a luminescence-based reporter assay with *Arabidopsis* seeds to detect chemical signals that can activate the KAI2 signaling pathway. We demonstrate that the *DLK2*:*LUC* assay can selectively and sensitively detect karrikins and a functionally similar synthetic strigolactone analog. Crucially we show that crude extracts from *Arabidopsis* leaves can also activate *DLK2*:*LUC* in a KAI2-dependent manner. Our work provides the first direct evidence for the existence of endogenous chemical signals that can activate the KAI2-mediated signaling pathway in *Arabidopsis*. This sensitive reporter system can now be used for the bioassay-guided purification and identification of putative endogenous KAI2 ligands or their precursors, and endogenous compounds that might modulate the KAI2 signaling pathway.

## Introduction

Karrikins (KAR) are potent compounds in wildfire smoke that stimulate germination of many plant species (Flematti et al., 2004), including *Arabidopsis thaliana* (Nelson et al., 2009). In *Arabidopsis*, response to KAR requires the F-box protein MORE AXILLARY GROWTH 2 (MAX2; Nelson et al., 2011) and the α/β-fold hydrolase KARRIKIN INSENSITIVE 2 (KAI2; Waters et al., 2012). Surprisingly, *kai2* and *max2* mutants are not only insensitive to KAR, but also show delayed germination and abnormal seedling growth phenotypes. Meanwhile, loss-of-function mutations in *SUPPRESSOR OF MAX2 1* (*SMAX1*) and its paralog *SMAX1-LIKE2* (*SMXL2*) induce constitutive KAR responses (Stanga et al., 2013, 2016). These mutant phenotypes suggest that the karrikin signaling pathway defined by KAI2, MAX2, and SMAX1/SMXL2 has endogenous functions in plant development that extend beyond mediating responses to KAR.

Karrikins are butenolide compounds structurally related to strigolactones (SLs), an endogenous set of butenolides that regulate shoot branching and other developmental processes (Waldie et al., 2014). Response to SL also requires MAX2 and a paralog of KAI2, namely DWARF14 (D14; Arite et al., 2009). Degradation of the SMAX1-LIKE proteins is induced by SL (Jiang et al., 2013; Zhou et al., 2013; Soundappan et al., 2015; Wang et al., 2015). As hydrolases, both KAI2 and D14 possess a catalytic triad (Ser, His, Asp) that is required for the function of both proteins (Hamiaux et al., 2012; Waters et al., 2015b). Three studies have demonstrated that D14 is activated by covalent modification of the catalytic triad following SL hydrolysis (Zhao et al., 2013; de Saint Germain et al., 2016; Yao et al., 2016), confirming D14 as a SL receptor (Hamiaux et al., 2012). Several reports have also demonstrated interaction of KAR with *Arabidopsis* KAI2 and its homologs (Guo et al., 2013; Kagiyama et al., 2013; Xu et al., 2016), but no covalent interaction has been reported. Additional butenolides such as synthetic SL isomer GR24*^ent^*^-5DS^ also activate KAI2 signaling, while mutation of the catalytic triad abolishes hydrolysis and signaling (Scaffidi et al., 2014; Waters et al., 2015b). As such, it is likely that KAI2 and D14 have similar modes of action as butenolide receptors. To date, no biosynthetic source of karrikins or karrikin-like compounds has been discovered. Instead, given the extensive similarities between KAI2 and D14 signaling and the fact that the SL biosynthetic pathway is not required for KAI2 signaling (Scaffidi et al., 2013), we have hypothesized that KAI2 may perceive an unknown endogenous KAI2 ligand (KL; Flematti et al., 2013). Recent genetic studies on KAI2 orthologs from parasitic plant species have provided indirect evidence to support this KL hypothesis (Conn et al., 2015; Conn and Nelson, 2016). Here we examine the KL hypothesis directly by asking whether the signaling pathway defined by KAI2-MAX2-SMAX1 can be activated by metabolites in plant extracts.

At the physiological level, KAR stimulates seed germination and inhibits hypocotyl elongation. However, other growth substances besides KL in plant extracts could potentially affect germination and seedling development in the same or opposite way as KL. Therefore, physiological responses to treatment with plant extracts could reflect a confounding and combinatorial effect of several active compounds, rather than any single class of compound.

Accordingly, we sought molecular responses to KL that are specifically dependent on the KAI2-MAX2-SMAX1 pathway. Among KAR-responsive transcripts, *D14-LIKE 2* (*DLK2*) transcription is strongly induced in a MAX2- and KAI2- or D14-dependent manner upon KAR or SL treatment (Waters et al., 2012; Scaffidi et al., 2014). Since D14 is comparatively weakly expressed in *Arabidopsis* seeds, *DLK2* serves as an explicit marker for KAI2-dependent signaling in seeds. Compared to wild type, *DLK2* transcripts are significantly less abundant in *kai2* and *max2* mutants (Waters et al., 2012), and more abundant in *smax1* and *smxl2* mutants (Stanga et al., 2013, 2016). These observations led us to investigate whether *DLK2* could serve as a specific indicator for activation of the KAI2-MAX2-SMAX1 pathway.

First we developed a rapid luminescence-based assay for up-regulation of *DLK2* in *Arabidopsis* seeds. We then established that the assay is sensitive and specific to KAR treatment compared to other known plant growth substances. Lastly we used the assay to examine *Arabidopsis* leaf extracts for KL activity.

## Materials and Methods

### Chemicals

Karrikins (KAR_1_, KAR_2_), GR24^5DS^, and GR24*^ent^*^-5DS^ were prepared as described (Flematti et al., 2007; Goddard-Borger et al., 2007; Scaffidi et al., 2014) and dissolved as 10 mM stock solutions in acetone. Epibrassinolide (Sigma E1641), gibberellic acid (GA_4_ from L. N. Mander, Australian National University), 3-indoleacetic acid (Sigma I2886), (+)-*cis*, *trans*-abscisic acid (AG Scientific A-1103) and (±)-jasmonic acid (Sigma J2500) were dissolved in acetone as 5, 10, 10, 10, and 50 mM stock solutions, respectively.

### *DLK2*:*LUC* Reporter Line Construction

The *DLK2* promoter sequence was defined as the 3566 bp of genomic sequence spanning the annotated transcriptional start site of *DLK2* (At3g24420) and 103 bp downstream of the annotated 3′ UTR of the preceding gene (At3g24430). We also included the *DLK2* 5′UTR (31 bp). This sequence was amplified by PCR using Phusion polymerase (New England Biolabs). Oligonucleotides were (5′–AAAAAAGCAGGCTCAAACGCGATAACCTTTTCA–3′) and MW446 (5′–CAAGAAAGCTGGGTGCTTAAGTACAAGAGTTTTG–3′); regions of homology to *Arabidopsis* genomic DNA are underlined. Gateway-compatible attB recombination sites were added in a further round of PCR and the resulting product cloned into pDONR207. This intermediate plasmid was recombined with the binary vector pHGWL7 (Karimi et al., 2002), inserting the *DLK2* promoter sequence upstream of firefly luciferase coding sequence.

The *DLK2:LUC* construct was introduced into *Arabidopsis* L*er* background by floral dipping. Primary transformants were selected on 20 μg ml^-1^ hygromycin B. Six lines that segregated 3:1 for hygromycin resistance were propagated to homozygosity and subsequently screened for LUC activity in response to KAR and racemic GR24. The most robustly responding line was then crossed with *kai2-2* (L*er*) (Waters et al., 2012) and experiments were performed on the F_3_ generation homozygous for both *kai2-2* and the *DLK2:LUC* transgene.

### Quantitative PCR

Twenty milligrams of *Arabidopsis* seeds were imbibed in 1 ml water supplemented with KAR or acetone for 48 h at 20°C under continuous light. RNA extraction and quantitative PCR was conducted as described (Waters et al., 2012). Oligonucleotides for *LUC* transcripts were 5′–ATTCTTTATGCCGGTGTTGG–3′ and 5′–TGTTGAGCAATTCACGTTCA–3′.

### Firefly Luciferase Standard Curve

Recombinant firefly luciferase in buffered aqueous solution (Sigma L9420) was diluted to 10^-8^g μl^-1^ with lysis solution (25 mM Tris-phosphate pH 7.8, 2 mM DTT, 2 mM 1,2-diaminocyclohexane-tetraacetic acid (DACTAA), 10% glycerol, 1% Triton X-100). A 10-fold dilution series in lysis solution was made, ranging from 10^-8^ g μl^-1^ to 10^-18^ g μl^-1^. Each luciferase stock solution was further diluted 1 in 20 with lysis solution and 20 μl of each dilution was transferred to a white opaque 96-well assay plate (Sigma-Aldrich, CLS3912) in triplicate. Triplicate lysis solution served as background control.

A POLARstar OPTIMA (BMG LABTECH) was used to measure luminescence. The injector was rinsed with 4.5 ml water, then primed with 1 ml Luciferase Assay Reagent (LAR; 15 mM K_2_PO_4_/KH_2_PO_4_ pH 7.8, 25 mM Gly-Gly, 4 mM EGTA, 15 mM MgSO_4_, 2 mM ATP, 1 mM DTT, 0.1 mM Coenzyme A, 120 μM luciferin) (Dyer et al., 2000). The instrument was programmed to inject 100 μl LAR (260 μl/second) one well at a time, to shake the assay plate for 5 s after each injection (1 mm shaking width, 600 rpm), and to measure luminescence signal for 5 s using the top optic (gain 4095). The assay plate was read from the lowest to the highest luciferase concentration, to avoid light contamination caused by higher-concentration luciferase samples. After measurements were completed, the mean luminescence signals produced by the background control lysis solution was subtracted from each luminescence reading produced by luciferase samples, to obtain the net reading for each sample.

### Assaying *DLK2*:*LUC* Activity

Dry *Arabidopsis* seeds (Landsberg *erecta*; 2.5 mg) were distributed with a home-made seed scoop into 1.2 ml tubes in eight-tube strips (Astral Scientific, I1720-00) held by a rack. The seeds were collected at the bottom by brief centrifugation. Compound stocks (1000-fold) in acetone were diluted in water and added to each tube (100 μl). The seeds were resuspended in treatment solutions by flicking the tubes. The seed tubes were incubated at 20°C under continuous light for between 24 and 72 h.

After incubation, the seed tubes were centrifuged at 4000 rpm for 5 min to collect seeds at the bottom. Treatment solution was removed from each tube using a multi-channel pipette set at 75 μl, with care taken not to remove any seeds. Two, 1 mm-diameter stainless steel balls, were distributed into each tube. Lysis solution (80 μl) was added to each tube. The seed tissues were ground in lysis solution using a mixer mill at 30/s for 1 min twice. The seed extracts were centrifuged at 4000 rpm for 10 min to pellet tissue debris. The supernatant (20 μl), containing extracted luciferase enzyme, was transferred to a white opaque 96-well assay plate (Sigma-Aldrich, CLS3912). Triplicate lysis solution served as background control.

Luminescence was measured as described in the previous section. The plate reading direction was perpendicular to the biological replicates loading direction, to avoid time-dependent bias.

The net readings were obtained as described in the previous section. The mean net luminescence reading was taken for mock and each treatment. Fold change in LUC activity was calculated by the following formula:

$$\text{Fold change in LUC activity = }\frac{\text{mean net luminescence reading }\left[\text{treatment}\right]}{\text{mean net luminescence reading }\left[\text{mock}\right]}$$

Standard errors of mean net luminescence readings were calculated and scaled to the fold change in LUC activity.

### Growth of *Arabidopsis* and Extraction of Metabolites

*Arabidopsis* L*er* seeds (0.6 ml) were sown directly on soil (peat:vermiculite:perlite 6:1:1) in 20 rectangular pots (Garden City Plastics, PUNSTX, volume 400 ml) distributed across two trays. The seeds were stratified for 3 days in the dark at 4°C, before being transferred to a climate-controlled growth room (8 h light/16 h dark photoperiod, 22°C light/16°C dark temperature cycle, 100–150 μmol m^-2^ s^-1^ PAR, 60% relative humidity). Rosette tissue from 7-week old plants prior to flowering was harvested, weighed (circa 200 g FW), and frozen in liquid nitrogen. Leaf tissue was ground in liquid nitrogen with a mortar and pestle, and extracted with 80% (v/v) methanol in water at 4°C overnight (10 ml per gram FW). The next day, the methanol/water extract was filtered with Whatman filter paper (18.5 cm, No. 4) to remove tissue debris. Methanol was removed under reduced pressure at 40°C. The remaining aqueous extract was diluted with water to 100 ml, and extracted with ethyl acetate (3 × 100 ml). The aqueous layer was concentrated under reduced pressure at 40°C to 10 ml and stored at -20°C. The combined ethyl acetate extract was evaporated to dryness under reduced pressure to give 0.8 g solid material. The ethyl acetate extract was re-constituted with 10 ml of purified water on a rotating wheel overnight at 4°C. A total of 0.1% of each extract (annotated as “stock”), and dilutions of 1/5 and 1/25 were tested with the *DLK2*:*LUC* assay.

### Statistical Analysis

Significance groupings were determined using one-way ANOVAs based on Honestly Significant Differences (HSD) Test. The analyses were performed in R Program v3.2.3, using the package “agricolae.”

## Results

### Development of the *DLK2:LUC* Reporter Assay

**FIGURE 1:**
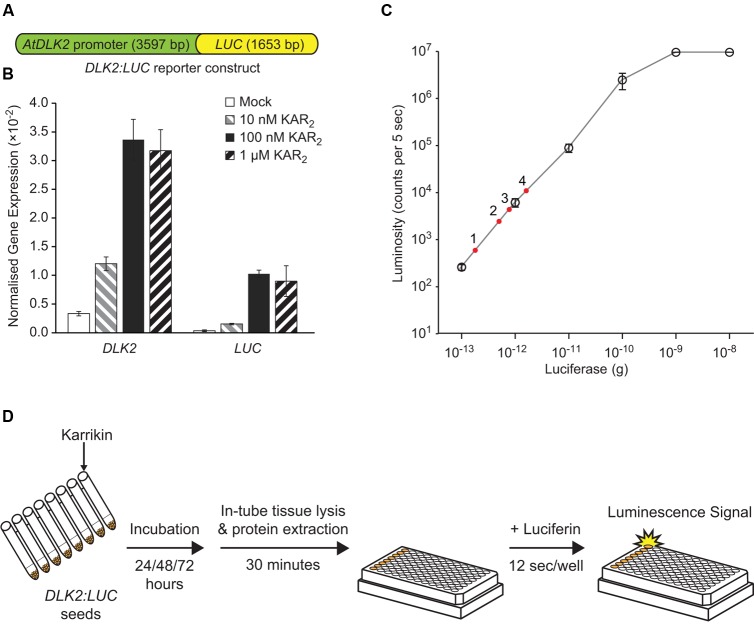
**Development of the *DLK2:LUC* reporter assay.** **(A)** Schematic representation of the *DLK2:LUC* reporter construct that was transformed to *Arabidopsis* wild-type L*er* (*DLK2:LUC* [L*er*]) and crossed into the *karrikin insensitive 2-2* (*kai2-2)* mutant background (*DLK2:LUC* [*kai2*]). The construct contains 3566 bp of intergenic sequence upstream of the transcriptional start site of *D14-LIKE 2* (*DLK2*, At3g24420), 31 bp of *DLK2* 5′UTR, and 1653 bp of *FIREFLY LUCIFERASE* (*LUC*) coding sequence. **(B)** Response of *DLK2* and *LUC* transcripts to karrikin treatments in *DLK2:LUC* reporter seeds. Transcript abundance was normalized to *CACS* (At5g46630). Error bars show standard errors (SE) with *n* = 3 batches of seeds. In this assay, neither *DLK2* nor *LUC* transcripts could be detected reliably in the *DLK2:LUC* [*kai2*] reporter seeds. **(C)** Standard curve of firefly luciferase enzymatic activity using purified firefly luciferase enzyme and D-luciferin substrate. Error bars show SE with *n* = 3 experimental replicates, where each dilution was measured with three technical replicates. The red dots represent a typical dataset where luciferase activity in *DLK2:LUC* [L*er*] is induced by (1) mock; (2) 10 nM KAR_2_; (3) 100 nM KAR_2_; (4) 1 μM KAR_2_ over 72 h. **(D)** Illustration of the *DLK2:LUC* assay procedure (illustration of the 96-well plate is adapted from Promega’s Technical Bulletin – Luciferase Assay System 12/11).

To measure *DLK2* expression while avoiding laborious RNA extraction and quantitative PCR steps, we fused the *DLK2* promoter (defined as 3566 bp upstream of the transcriptional start site, plus 31 bp of 5′UTR) to the firefly luciferase (*LUC*) gene to make the reporter *DLK2*:*LUC* (**Figure 1A**). We chose such a long intergenic region to avoid excluding potential upstream regulatory elements. We reasoned that, in a wild type background, KAR would activate *LUC* gene expression and induce luciferase activity, but that this response would be absent in *kai2* mutants. Accordingly we generated transgenic *Arabidopsis* expressing the reporter construct in L*er* ecotype, and then crossed a suitably responding transgenic line with *kai2-2*. We first tested induction of *LUC* gene expression upon KAR treatments using quantitative RT-PCR, in comparison with the endogenous *DLK2* gene in *Arabidopsis* seeds. In imbibed seed, *DLK2* transcripts increase in response to KAR via KAI2, while signaling via D14 is low or absent (Waters et al., 2012). We found that both *DLK2* and *LUC* transcripts were induced by KAR_2_ treatments (**Figure 1B**). While levels of *LUC* transcripts were lower than *DLK2* transcripts (relative to *CACS* reference transcripts), the patterns of induction in response to KAR_2_ were very similar.

We then adopted a luciferase assay system in a 96-well plate format to increase throughput. We generated an enzymatic activity standard curve for the assay system using recombinant firefly luciferase standards (**Figure 1C**). In our hands, the system detection limit is 10^-13^ g of luciferase, with a linear response between 10^-13^ g to 10^-9^ g of luciferase enzyme, which compares favorably with published data (Dyer et al., 2000).

We used a cell-free luciferase detection method to avoid blocking of luminescence signals by seed tissues (**Figure 1D**). Compounds for treatment were dissolved in water and applied to the *DLK2*:*LUC* reporter seeds. After imbibition, cell lysate was prepared from the seeds, and luminescence was measured by an automated plate reader. The luminescence signal produced by a particular treatment was expressed relative to the signal produced by seeds treated with water alone (mock treatment).

### Validation of the *DLK2:LUC* Reporter Assay

**FIGURE 2:**
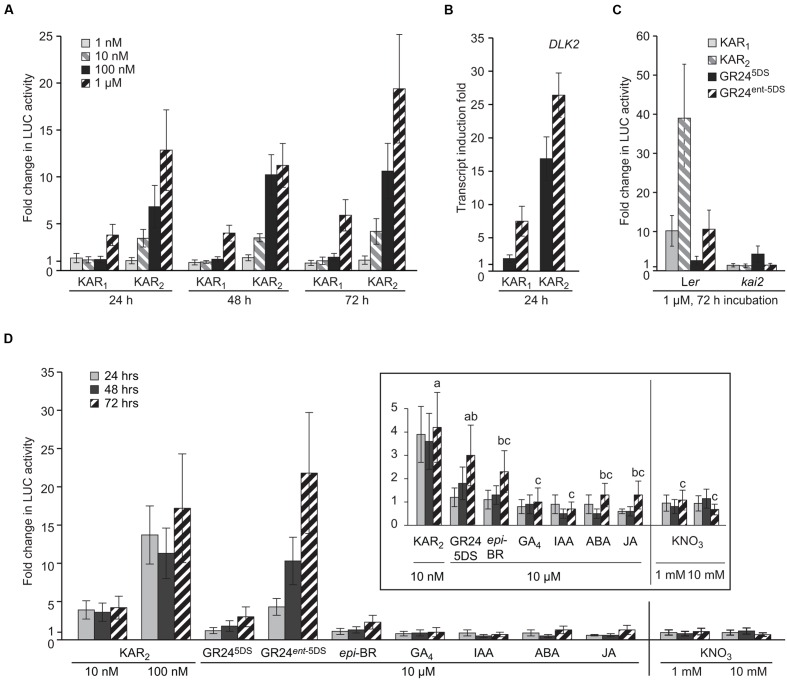
***DLK2:LUC* Specifically Responds to KAI2 Substrates.** **(A)** Sensitivity of the *DLK2:LUC* assay to KAR_1_ and KAR_2_ at a range of concentrations over 24, 48, and 72 h. Data are expressed as fold change in LUC activity relative to a mock sample of seeds treated with water for the same duration. **(B)** Sensitivity of *DLK2* transcripts to KAR_1_ and KAR_2_ at a range of concentrations over 24 h in L*er* seeds. Data are expressed as fold change relative to mock sample of seeds treated with water for the same duration. **(C)** Specificity of the *DLK2:LUC* assay toward karrikins and strigolactones in L*er* and *kai2* backgrounds treated for 72 h. **(D)** Specificity of the *DLK2:LUC* assay toward strigolactone analogs, selected plant hormones, and the germination stimulant KNO_3_. Vertical line indicates separate experiments. Inset shows the same data scaled for smaller fold changes. Shared lower case letters indicate no significant difference between 72 h treatments, and “c” indicates no significant difference from mock treatment (ANOVA; *P* < 0.05). In all charts, error bars depict SE, *n* = 8 replicates.

To demonstrate the sensitivity of the *DLK2:LUC* assay, we applied a concentration gradient of the two karrikins KAR_1_ and KAR_2_ over a time-course of 72 h to the *DLK2*:*LUC* [L*er*] reporter seeds. Using this method, KAR response can be detected after 24 h (**Figure 2A**). The system is substantially more sensitive to KAR_2_ treatment since 10 nM KAR_2_ induced a fourfold change in activity within 24 h, while 1 μM KAR_1_ was necessary for a similar response. This preference for KAR_2_ is consistent with multiple responses in *Arabidopsis* (Nelson et al., 2011; Waters et al., 2012, 2015a), as well as for endogenous *DLK2* transcripts themselves (**Figure 2B**). To demonstrate the specificity of the *DLK2*:*LUC* assay system in differentiating responses to KAR and SL, we applied KAR_1_, KAR_2_, and two enantiomers of the synthetic SL analog GR24 to *DLK2*:*LUC* [L*er*] and *DLK2*:*LUC* [*kai2*] seeds for 72 h. GR24^5DS^ is a synthetic SL with stereochemistry consistent with natural SLs that act preferentially through D14, whereas its non-naturally configured enantiomer GR24*^ent^*^-5DS^ operates largely via KAI2 (Scaffidi et al., 2014; Waters et al., 2015a). As expected, *DLK2*:*LUC* [L*er*] seeds responded strongly to KAR_1_, KAR_2_ and GR24*^ent^*^-5DS^, but only marginally to GR24^5DS^ (**Figure 2C**). Importantly, the responses to KAR_1_, KAR_2_, and GR24*^ent^*^-5DS^ were eliminated in the *DLK2*:*LUC* [*kai2*] seeds. These data demonstrate that induction of LUC activity is ligand-specific and KAI2-dependent.

To investigate further the specificity of the *DLK2*:*LUC* assay system, we applied a variety of plant hormones [epibrassinosteroid (*epi*-BR); gibberellic acid (GA_4_); auxin (IAA); abscisic acid (ABA); jasmonic acid (JA)] and the germination stimulant KNO_3_ over a period of 72 h. We also included the two enantiomers of GR24. We found that while 100 nM KAR_2_ and 10 μM GR24*^ent^*^-5DS^ induced a 10-fold increase in luciferase activity after 48 h, the other tested compounds were essentially inactive (**Figure 2D**). Therefore *DLK2:LUC* activity is not affected by these known plant hormones or KNO_3_. There was a limited response to GR24^5DS^ after 72 h, which may indicate increasing expression of the SL receptor D14 after prolonged seed imbibition.

### Detection of *DLK2:LUC* Induction Activity in *Arabidopsis* Metabolites Extracts

Having validated the sensitivity and specificity of the *DLK2:LUC* assay, we used it to test whether compounds extracted from *Arabidopsis* tissue could activate the KAI2-MAX2-SMAX1 signaling pathway. We reasoned that leaf material would be a good source of KL because this was a simple way to gather relatively large amounts of tissue. In addition, the defective leaf development phenotype of *kai2* mutants indicated that KAI2-dependent signaling is active beyond the seed and seedling stages where large amounts of material would be more difficult to obtain.

**FIGURE 3:**
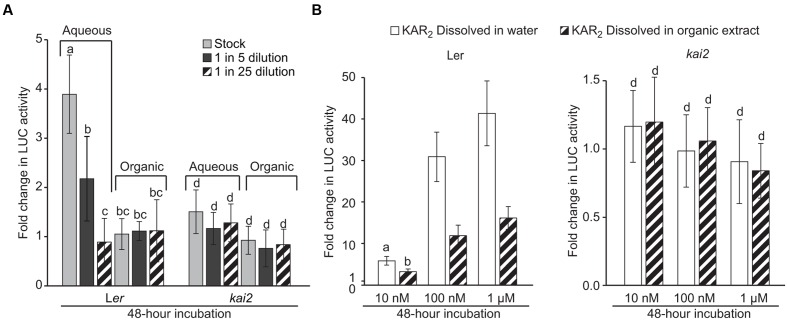
***DLK2:LUC* activity is induced by *Arabidopsis* leaf extracts.** **(A)** Activity of *Arabidopsis* leaf extracts in inducing *DLK2:LUC* expression in a KAI2-dependent manner. Extracts were separated into aqueous and organic fractions and applied separately. Stock treatment is equivalent to 0.2 g of rosette tissue from 7-week old *Arabidopsis* plants grown under an 8 h day/16 h night photoperiod. Error bars depict SE, *n* = 3 replicates. **(B)** Activity of KAR_2_ standard dissolved either in water or in the organic fraction of the *Arabidopsis* leaf extract. The concentration of organic extract is equivalent to the stock treatment concentration in **(A)**. Error bars depict SE, *n* = 8 replicates. Shared lower case letters indicate no significant difference between treatments within the same genotype. “c” and “d” indicate no significant difference from mock-treated *DLK2:LUC* [L*er*] and *DLK2:LUC* [*kai2*] seeds, respectively. (ANOVA; *P* < 0.05).

We extracted metabolites from *Arabidopsis* rosettes with 80% (v/v) methanol in water. After removing the methanol by evaporation, we added ethyl acetate and partitioned the crude extract into two layers: aqueous (water) and organic (ethyl acetate). The partitioning method was originally optimized to extract KAR_1_ from aqueous solutions. We applied a dilution series of each extract to the *DLK2*:*LUC* [L*er*] and *DLK2*:*LUC* [*kai2*] reporter seeds. We found that unknown metabolites in the aqueous layer increased *DLK2* expression fourfold within 48 h in a KAI2-dependent manner, whereas metabolites in the organic layer were inactive in this assay (**Figure 3A**). As such, we infer that compounds present in the *Arabidopsis* leaf extract can activate the KAI2-MAX2-SMAX1 signaling pathway. To our surprise, the active compound(s) appeared to be water-soluble and inefficiently extracted into ethyl acetate. This is in marked contrast to KAR_1_ and SLs, which are usually extracted into organic solvents such as ether or ethyl acetate (Yasuda et al., 2003; Flematti et al., 2004).

We considered that the organic layer might in fact be active, but also might contain inhibitors that prevent the activation of *DLK2:LUC*. To detect such inhibitors, we compared the relative activity of KAR_2_ dissolved in water versus that of KAR_2_ dissolved in the organic layer (**Figure 3B**). We found that KAR_2_ dissolved in the organic layer induced *DLK2:LUC* to approximately half the level of KAR_2_ dissolved in water, suggesting that inhibitors were likely present but were insufficient to completely suppress *DLK2:LUC* activation, even at low KAR_2_ concentration. Because we only observed activity in the aqueous fraction (**Figure 3A**), it is unlikely that there was appreciable activity in the organic layer that was suppressed by inhibitors. We cannot prove conclusively the absence of any activity in the organic layer because any extraction or recovery process might exclude some compounds. However, on the basis of these results, we conclude that the active compound(s) is primarily water-soluble.

## Discussion

The classical plant hormones were discovered through their bioactivities when applied exogenously. For example, the observation that extracts of senescent leaf tissues accelerated abscission of de-bladed young leaves led to the hypothesis of a new hormone (Osborne, 1955), which was later identified as abscisic acid (Liu and Carnsdagger, 1961). In this case, a clearly defined source of bioactivity (senescent leaves) contained the hypothetical hormone, while a biological response (abscission) indicated the effect of the hormone. In contrast, the hypothesis of a KAI2 ligand (KL) is distinct in nature, because rather than starting from an observed bioactivity, the existence of a hormone is inferred from genetic analyses and by analogy to SL signaling. Therefore, to isolate KL, it is necessary first to identify a source of bioactivity, and then determine a specific biological response that indicates the presence of KL bioactivity in the source. Here, we identified *Arabidopsis* leaf extracts as a source for KL bioactivity, and *DLK2* induction as the specific biological response. The resulting reporter system allows the source-response relationship to be assayed readily. The approach adopted here mirrors that of Adhikari et al. (2013), who used a reporter system to establish both the presence and chemical nature of the ‘bypass’ signal that controls shoot growth, although its identity is yet to be determined.

The main evidence for KAI2 being the receptor for an unknown endogenous compound is fivefold. First, a KAI2-like protein is the evolutionary ancestor of D14, the strigolactone receptor (Delaux et al., 2012; Waters et al., 2012, 2015b). Second, KAI2-dependent signaling requires the catalytic triad as does D14, and this requirement is evolutionarily conserved between lycophytes and angiosperms (Waters et al., 2015a,b). Third, some divergent KAI2 homologs in parasitic weeds within the Orobanchaceae have become specialized for strigolactone perception, while more evolutionarily conserved homologs have retained a strigolactone-independent function similar to that of AtKAI2 (Conn et al., 2015; Toh et al., 2015; Conn and Nelson, 2016). Fourth, both KAI2- and D14-dependent signaling operates via the same family of SMXL repressor proteins (Jiang et al., 2013; Stanga et al., 2013, 2016; Zhou et al., 2013; Soundappan et al., 2015; Wang et al., 2015). Finally, *kai2* and *max2* mutants of *Arabidopsis* both share seed germination and seedling morphogenesis phenotypes that are opposite to the effects of karrikin treatment and that are not found in strigolactone mutants (Nelson et al., 2011; Waters et al., 2012). In agreement with these compelling molecular-genetic evidence, here we have demonstrated that compounds extracted from plant tissue can activate KAI2-dependent signaling, further supporting the existence of KL.

The *DLK2:LUC* assay we describe here can be scaled up and adapted to isolate the active compound(s), and potentially identify KL. There are at least three major challenges to doing so. First, the response of *DLK2:LUC* to leaf extracts is comparable to just 10 nM KAR_2_, and KL is presumably a more efficient KAI2 ligand than KAR_2_. This observation suggests that the active compound(s) is presumably very low in abundance, necessitating large-scale growth of source material. The structural elucidation of the gibberellin GA_32_, for example, involved the isolation of 38 mg from 35 kg of peach seeds, themselves isolated from one ton of fruit (Yamaguchi et al., 1970; Mander, 2003). As a further example, the isolation of ABA from cotton leaf petioles required 10 kg of dry plant material to isolate just 1 mg (Liu and Carnsdagger, 1961). Such low yields probably preclude the use of *Arabidopsis* leaves as a source, necessitating a hunt for a richer or commercially available source. The second challenge may involve the isolation of potentially unstable compounds (e.g., if KL is similar to strigolactones, which hydrolyse in water) through several rounds of separation. Investigation of different solvents, extraction techniques and separation methods will assist in solving this problem. Finally, the reporter assay itself could be refined to improve specificity and sensitivity. For example, use of a *d14* mutant background could exclude false positives from strigolactones, which may activate the system at later stages of seed germination (**Figure 2D**). Conceivably, sensitivity improvements could result from an optimized, synthetic promoter consisting of concatemerized “KAR-response” elements identified from the *DLK2* promoter.

Based on the broad similarities between karrikins, SLs and their respective receptors, we would expect KL to be a hydrophobic butenolide compound or group of compounds. However, it should be noted that the active compound(s) detected by this technique might not be the direct ligand(s) of KAI2. Although the assay indicates KAI2-specific induction of *DLK2* expression, it does not differentiate KL from other signals upstream of KAI2. Potentially, the activity observed in this assay – which was unexpectedly water-soluble – might originate from a biosynthetic precursor of KL, or a stimulator of KL biosynthesis. Nevertheless, identifying any such chemical interactors of the KAI2-MAX2-SMAX1 pathway would greatly enhance our understanding of the endogenous functions of the pathway. Discovering the identity of KL would be a major advance for plant hormone biology. Furthermore, KL could be beneficial as an agrichemical in applications where KAI2-mediated control of seed germination and early seedling establishment is critical.

## Author Contributions

YS, GF, SS, and MW designed the research. YS and MW performed the research. YS, GF, SS, and MW analyzed the data and wrote the manuscript.

## Conflict of Interest Statement

The authors declare that the research was conducted in the absence of any commercial or financial relationships that could be construed as a potential conflict of interest.
